# Synchronous occurrence of gastrointestinal stromal tumor, pancreatic intraductal papillary mucinous neoplasm, and intrahepatic cholangiocarcinoma: Case report

**DOI:** 10.1097/MD.0000000000029460

**Published:** 2022-07-15

**Authors:** Qiaoru Hou, Wenjun Zhang, Jiazeng Niu, Minghua Tian, Jie Liu, Linyang Cui, Yingming Li

**Affiliations:** a Diagnostic Imaging Center of Weihai Central Hospital, Weihai, Shandong, China; b Hepatological Surgery Department of Weihai Central Hospital, Weihai, Shandong, China; c Obstetrics Department of Weihai Central Hospital, Weihai, Shandong, China; d Pathology Department of Weihai Central Hospital, Weihai, Shandong, China.

**Keywords:** concurrent tumors, GIST, imaging and pathologic diagnosis

## Abstract

**Rationale:**

Gastrointestinal stromal tumor (GIST) is the most common primary mesenchymal tumors in gastrointestinal tract. Synchronous occurrence of GIST and tumors in other organs is rare. We first report an exceedingly rare case of synchronous occurrence of gastric GIST, pancreatic intraductal papillary mucinous neoplasm (IPMN) and intrahepatic cholangiocarcinoma.

**Patient concerns:**

A 70-year-old male presented to our hospital because of abdominal pain and dyspepsia. Tumor markers and liver function were abnormal. Abdomen computed tomography showed concurrent tumors in stomach, pancreas, and liver.

**Diagnosis:**

Pathology confirmed synchronous occurrence of gastric GIST, pancreatic IPMN and intrahepatic cholangiocarcinoma.

**Interventions:**

Mass excision, partly gastrectomy, wedge resection of VIII liver segments, and pancreatic-oduodenectomy were performed.

**Outcomes:**

During the 18-month follow-up, both laboratory tests and computed tomography examination revealed no sign of recurrence or metastasis. Currently, the patient is free of clinical symptoms such as abdominal discomfort, jaundice, and fever.

**Conclusion:**

As yet, no cases simultaneously with gastric GIST, pancreatic IPMN and intrahepatic cholangiocarcinoma have been described in literatures. This report increases the knowledge to avoid misdiagnosis and delayed therapy for coexistence of the described 3 types of neoplasm.

## 1. Introduction

Gastrointestinal stromal tumor (GIST), though being rare tumor, is the most common mesenchymal tumor in the gastrointestinal tract with an estimated incidence of 14.5 per million.^[1]^ GIST is derived from the interstitial cells or their stem cell-like precursors.^[2]^ The incidence of coexisted GIST with other tumors is relatively high, ranging from 4.5% to 33%.^[3]^ Recently, synchronous occurrence of GIST and tumors in other organs has an increased incidence, and then increase the mortality. Gastric carcinoma was the most common synchronous tumor,^[4]^ followed by breast and prostate carcinoma, renal cell carcinoma, desmoid-type fibromatosis, leukemia, colorectal cancer.^[5–8]^ In this paper, we report a case of a 70-year-old male patient with synchronous occurrence of gastric GIST, pancreatic intraductal papillary mucinous neoplasm (IPMN) and intrahepatic cholangiocarcinoma, which is the first case ever reported.

## 2. Informed Consent

Written informed consent was obtained from the patient for publication of the case details and accompanying images.

## 3. Case Description

A 70-year-old male visited our department with abdominal pain and dyspepsia for 10 days. He has no previous surgeries or family history of digestive diseases. Physical examination revealed mild epigastric tenderness with no palpable abdominal mass. No abnormalities in routine blood biochemical examination were evident. Tumor markers (including CEA and CA19-9) were slightly higher than the normal level while alpha fetal protein was within the normal range. Liver function tests revealed obviously elevated glutamic-pyruvic transaminase (148 IU/L), glutamic oxalacetic transaminase (301 IU/L), and slightly elevated glutamyl transpeptidase (94 IU/L), direct bilirubin (10.3 mg/dL). Albumin, alkaline phosphatase, and cholinesterase were lower than normal levels. It is worth mentioning that amylase was significantly elevated (3200 IU/L).

**Figure 1.**
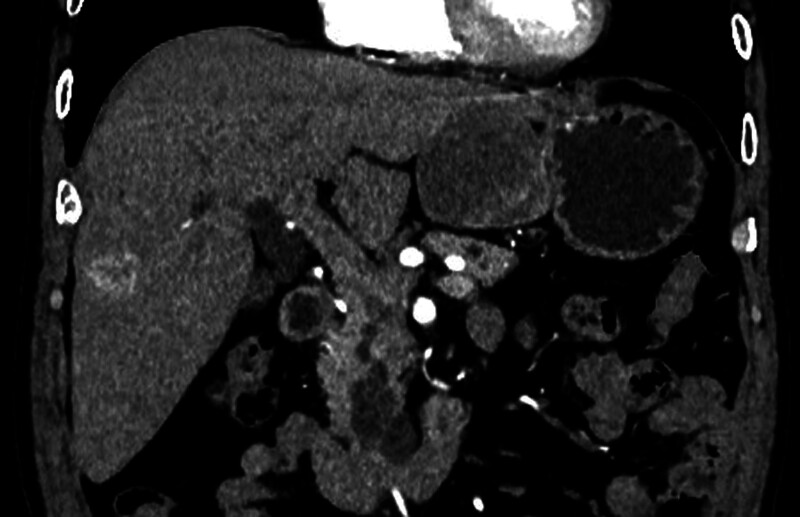
CT shows gastric GIST located in the lesser curvature of the stomach, intrahepatic cholangiocarcinoma located in VIII liver segments and pancreatic IPMN located in the head of the pancreas. CT = computed tomography, GIST = gastrointestinal stromal tumor, IPMN = intraductal papillary mucinous neoplasm.

**Figure 2.**
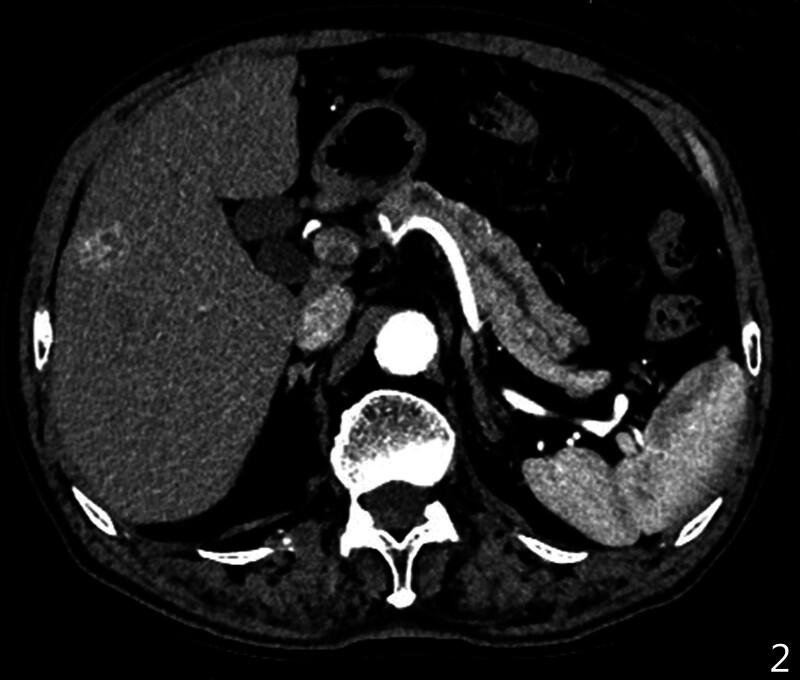
CT shows the dilated pancreatic duct and intrahepatic cholangiocarcinoma located in VIII liver segments. CT = computed tomography.

Computed tomography (CT) scan revealed a lower-density mass located in the lesser curvature of the stomach with heterogeneously mild enhancement. This mass was highly suspected to be a GIST (Fig. 1). Moreover, an ill-defined low-density lesion was identified at VIII liver segments. This lesion showed evidently enhancement at arterial phase and delayed attenuation similarly to aorta (Figs. 1 and 2 ). CT also demonstrated a dilated pancreatic duct and multiple cystic low-density lesions at the head of the pancreas with no obvious enhancement (Figs. 1 and 2). Significantly enlarged lymph nodes were not observed in the abdominal cavity.

**Figure 3.**
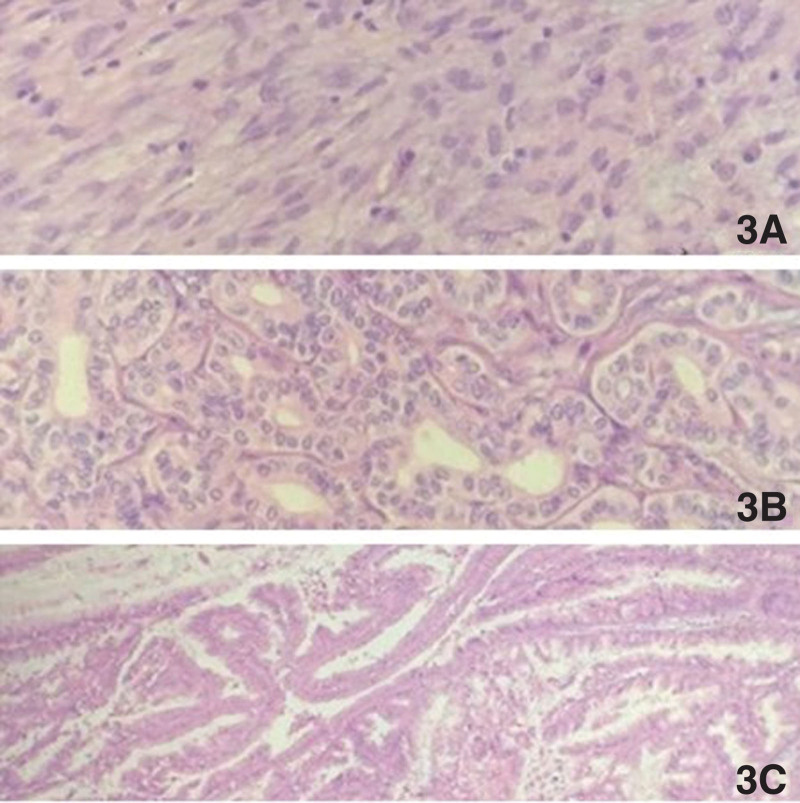
Histopathological results: (A) gastric GIST was composed of spindle cells arranged in a fascicular-like manner with visible paracentric vacuoles. (B) Intrahepatic cholangiocarcinoma was consisted of unequal-sized and heterogenic glands. (C) Pancreatic IPMN: obvious mucous columnar epithelial hyperplasia in papillary arrangement with cells in medium atypia hyperplasia (hematoxylin eosin stain, ×400). GIST = gastrointestinal stromal tumor, IPMN = intraductal papillary mucinous neoplasm.

Two days later, gastric mass excision, partly gastrectomy, wedge resection of VIII liver segments, and pancreatic-oduodenectomy were performed. Biopsy revealed a 6 × 5 × 4.5 cm GIST with <5 mitoses/50 high power fields (HPF) in moderate histologic grade. According to the risk stratification of National Institutes of Health consensus criteria, the GIST was classified as intermediate risk (size: 5–0 cm; mitotic count: 5/50 HPF).^[9]^ Immunohistochemical results: CD117(+), DOG1(+), Ki-67(+, 2%), SMA(−), desmin(−), S-100(−). Histological examination of the specimens showed that it was composed of spindle cells arranged in a fascicular-like manner with visible paracentric vacuoles (Fig. 3A). Tumor in the right lobe of liver was proved to be highly differentiated cholangiocarcinoma, which consisted of unequal-sized and heterogenic glands. The cells in gland were moderately atypical with significant interstitial fibrous reaction (Fig. 3B). The multiple cystic lesions at the head of the pancreas were pathologically confirmed to be pancreatic IPMN with active cell growth and suspected infiltration of the wall. Histopathological analysis revealed obvious mucous columnar epithelial hyperplasia in papillary arrangement with cells in medium atypia hyperplasia (Fig. 3C). Ten lymph nodes were evaluated with no signs of malignancy.

During the 18-month follow-up, both laboratory tests and CT examination revealed no sign of recurrence or metastasis. Currently, the patient is free of clinical symptoms such as abdominal discomfort, jaundice, and fever.

## 4. Discussion

GIST is the most common primary mesenchymal tumors in gastrointestinal tract with an annual incidence of 11 to 19.6 cases per 100,000 individuals.^[10]^ GIST was defined in 1983,^[11]^ and include tumors in gastrointestinal tract that cannot be classified as either smooth muscle or neurogenic in origin. GIST is derived from the interstitial cells, which are KIT-positive, pacemaker cells that regulate peristalsis and have varying immunophenotypic and ultrastructural features of both smooth muscle and neural differentiation. KIT protein activation by mutations may result in interstitial cells proliferation and GIST.^[12]^ About 5% of GIST is associated with syndromes or specific inheritable mutations, such as neurofibromatosis type 1, Carney triad, Carney dyad, and familial GIST.^[13]^ The mutations of KIT, PDGFRA, and succinate dehydrogenase genes have been described in familial GIST.^[14]^ The majority of GIST appear to occur sporadically, with most cases in the stomach (60%–70%).^[15]^ Gastric GIST can be incidentally detected or generally manifested with non-specific symptoms, such as nausea, vomiting, and abdominal pain.^[3]^ The low rate of preoperative diagnostics can be ascribed to small sizes and intramural localizations of the tumors.^[6]^

The probability of coexisted GIST with other neoplasms is ranging from 4.5% to 33%. Imaging examination can early detect coexisting neoplasms and provide key information for localization and diagnosis. Pathologic diagnosis plays a role part in providing early diagnosis at the early stage, so as to enable early management and avoid complications. CT manifestations of GIST are generally a well-defined exophytic mass, while intraluminal or dumb bell-shaped may occasionally occur. Small tumors are submucosal or endoluminal polypoid masses with homogenous contrast enhancement. While large tumors show irregular lobulated margins, mucosal ulceration, central necrosis, hemorrhage, cavitation, and heterogeneous enhancement. Large size, hepatic metastasis, and presence of wall invasion often suggest a high-grade GIST and predict poor outcome.^[16]^ Tumor with the size >5 cm are associated with greater risks of metastasis, commonly metastasizes to the liver or peritoneum.^[6]^ Most metastases are hypervascular on the early phase of dynamic imaging compared to the surrounding tissue.^[17]^

GIST display a wide variety of histopathological features, making it difficult to confirm with a definite diagnosis.^[7]^ Pathologic diagnosis is based on both unique microscopic features (fusiform, epithelioid, or mixed type) and immunohistochemical techniques (CD117, CD34, actin, desmin, S-100, and Ki-67) with counting of the number of mitoses per 50 HPF.^[18]^ Different types of mutations can be found in KIT and PDGFRA genes encoding a receptor tyrosine kinases type III (RTC). GIST in stomach is less aggressive than those in intestine. A low mitotic rate, <5 mitoses/50 HPF, most frequently indicates benign clinical behaviors, while GIST with more than 50 mitoses/50 HPF is provided with a high degree of malignity.^[19]^

Pancreatic IPMN and intrahepatic cholangiocarcinoma were more common in older men. Intrahepatic cholangiocarcinoma have many pathogenic factors, such as primary sclerosing cholangitis, ulcerative colitis, Caroli disease, intrahepatic bile duct stones, infection. Hepatic metastasis is one of the most common sites of metastases from GIST. And once that happens, the prognosis is almost invariably poor.^[9]^ For this case, pathological results confirmed the diagnosis of cholangiocarcinoma, ruled out intrahepatic metastasis of GIST. The synchronous or metachronous occurrence of GIST in patients with a background of other neoplasias must always be taken into consideration.^[20]^ GIST and other cancers have many significant associations, and GIST population has an increased cancer risk. The increased occurrence of other cancers was specific to the anatomic site and the histological type, and it had a temporal relation with the GIST diagnosis. The standardized incidence ratios of hepatobiliary adenocarcinoma and pancreatic adenocarcinoma risk after GIST were 3.10% and 2.03%.^[21]^ Although the study did not address the standardized incidence ratios of IPMN, a fraction of IPMN can develop into pancreatic adenocarcinoma. The incidence of hepatobiliary adenocarcinoma and pancreatic adenocarcinoma could significantly elevate after GIST. The period most frequently occurred additional cancers was within 1 year before and after the GIST. We boldly hypothesized that GIST may play a role in the occurrence of intrahepatic cholangiocarcinoma and IPMN.

Surgical resection is the standard treatment of GIST. Surgery could remove the GIST completely, and the risk stratification of this case was intermediate risk. Targeted medical therapy by tyrosine kinase inhibitors is recommended only for GIST that is marginally resectable or resectable but with a risk of significant morbidity.^[22]^ So the doctors and patient unanimous decided that targeted medical therapy was not given. According to the American College of Gastroenterology Guideline, resection was recommended for patients with main duct IPMN considered to be at acceptable surgical risk. Observation only for branch duct IPMN ≤2 cm without mural nodule.^[23]^ For intrahepatic cholangiocarcinoma, surgical resection remains the mainstay of potentially curative treatment. For patients with advanced-stage or unresectable cholangiocarcinoma, locoregional and systemic chemotherapeutics are the primary treatment options.^[24]^ Regular check was requested for every 3 to 6 months.

This study is limited due to the patient’s refusal for performance of genetic investigations. Therefore, it is unable to prove a specific genetic disorder that may provoke the concomitant appearance of these neoplasms.

As yet, no cases simultaneously with gastric GIST, pancreatic IPMN and intrahepatic cholangiocarcinoma have been described in literatures. Proper identification and description of the link between the 3 have a great significance in screening, prevention, diagnosis, and treatment disease. This report raises awareness to avoid misdiagnosis and delayed therapy for coexistence of the described 3 types of neoplasm, and provides a reference to capture the potential syndromes. It is hoped that this case will be valuable in the future investigations about the genesis, diagnosis, and treatment of such types of tumors.

## Correction

When originally published, the authors for reference 6 appeared incorrectly as “Marina M, Vladimir J, Marina P, et al.” and have been corrected to “Markovic M, Jurisic V, Petrovic M et al.”
